# Mobile Tuberculosis Treatment Support Tools to Increase Treatment Success in Patients with Tuberculosis in Argentina: Protocol for a Randomized Controlled Trial

**DOI:** 10.2196/28094

**Published:** 2021-06-21

**Authors:** Sarah Iribarren, Hannah Milligan, Kyle Goodwin, Omar Alfonso Aguilar Vidrio, Cristina Chirico, Hugo Telles, Daniela Morelli, Barry Lutz, Jennifer Sprecher, Fernando Rubinstein

**Affiliations:** 1 Biobehavioral Nursing and Health Informatics University of Washington Seattle, WA United States; 2 National Tuberculosis Control Program Region Five, Buenos Aires Argentina Buenos Aires Argentina; 3 Institute of Clinical Effectiveness and Healthcare Policy Buenos Aires Argentina; 4 Department of Bioengineering University of Washington Seattle, WA United States

**Keywords:** tuberculosis, disease management, infectious disease, mHealth, digital health, direct drug metabolite test, mobile phone

## Abstract

**Background:**

Tuberculosis (TB) is an urgent global health threat and the world’s deadliest infectious disease despite being largely curable. A critical challenge is to ensure that patients adhere to the full course of treatment to prevent the continued spread of the disease and development of drug-resistant disease. Mobile health interventions hold promise to provide the required adherence support to improve TB treatment outcomes.

**Objective:**

This study aims to evaluate the effectiveness of the TB treatment support tools (TB-TSTs) intervention on treatment outcomes (success and default) and to assess patient and provider perceptions of the facilitators and barriers to TB-TSTs implementation.

**Methods:**

The TB-TSTs study is an open-label, randomized controlled trial with 2 parallel groups in which 400 adult patients newly diagnosed with TB will be randomly assigned to receive usual care or usual care plus TB-TSTs. Participants will be recruited on a rolling basis from 4 clinical sites in Argentina. The intervention consists of a smartphone progressive web app, a treatment supporter (eg, TB nurse, physician, or social worker), and a direct adherence test strip engineered for home use. Intervention group participants will report treatment progress and interact with a treatment supporter using the app and metabolite urine test strip. The primary outcome will be treatment success. Secondary outcomes will include treatment default rates, self-reported adherence, technology use, and usability. We will assess patients’ and providers’ perceptions of barriers to implementation and synthesize lessons learned. We hypothesize that the TB-TSTs intervention will be more effective because it allows patients and TB supporters to monitor and address issues in real time and provide tailored support. We will share the results with stakeholders and policy makers.

**Results:**

Enrollment began in November 2020, with a delayed start due to the COVID-19 pandemic, and complete enrollment is expected by approximately July 2022. Data collection and follow-up are expected to be completed 6 months after the last patient is enrolled. Results from the analyses based on the primary end points are expected to be submitted for publication within a year of data collection completion.

**Conclusions:**

To our knowledge, this randomized controlled trial will be the first study to evaluate a patient-centered remote treatment support strategy using a mobile tool and a home-based direct drug metabolite test. The results will provide robust scientific evidence on the effectiveness, implementation, and adoption of mobile health tools. The findings have broader implications not only for TB adherence but also more generally for chronic disease management and will improve our understanding of how to support patients facing challenging treatment regimens.

**Trial Registration:**

ClinicalTrials.gov NCT04221789; https://clinicaltrials.gov/ct2/show/NCT04221789.

**International Registered Report Identifier (IRRID):**

DERR1-10.2196/28094

## Introduction

### Background

Tuberculosis (TB) is among the leading causes of death globally, surpassing HIV and malaria, even though most cases are preventable and curable. In 2019, the World Health Organization (WHO) reported a global TB incidence of 10 million, with approximately 1.4 million deaths [1]. To address the global health emergency that TB represents, the WHO End TB Strategy has set goals to reduce deaths and incidence rates by 95% and 90%, respectively, by 2035, relative to 2015 [2]. A critical challenge to meet these targets is to ensure that patients adhere to the full course of treatment to prevent the continued spread of TB and the development of drug-resistant disease. The WHO and others recognize that current strategies to ensure treatment success are insufficient to meet the goal of TB elimination in this century, and new treatment strategies are needed [3-5]. Interest in mobile health (mHealth) or digital health interventions to address these challenges and support patients throughout their treatment is growing, yet rigorous research is needed to evaluate these solutions in diverse settings and varying models of care [6-8].

Of the mHealth approaches under investigation for TB adherence monitoring, drug metabolite testing has been identified as the most promising, ethical, accurate, and least intrusive and stigmatizing strategy compared with other mobile solutions (eg, video observation, medication bottles containing sim card, and ingestible sensors), yet its potential remains largely unexplored [9]. Similarly, mobile apps can provide personalized treatment supervision, increase patients’ self-management, and improve patient-provider communication by offering advanced functionalities for patient support and monitoring [10]. However, most apps are consumer facing, whereas health care systems are provider facing; thus, when digital health tools are not connected to systems and human support, they are unlikely to be effective [11]. In systematic reviews of TB-related apps in the marketplace, most apps either targeted health care workers (eg, dosing calculations) or provided general TB information; few of them targeted patients and none supported TB patient engagement in their own care (eg, self-tracking and side effect monitoring) [12,13]. Furthermore, none of them were reported to have been developed and tested for Latinos or Spanish speakers [12].

This protocol builds on preliminary work to develop and refine a patient-centered tuberculosis treatment support tools (TB-TSTs) based on patient and expert feedback through focus groups, field testing, and pilot testing (K23NR017210, primary investigator: Iribarren) and to test the TB-TSTs (R01AI147129, multiple primary investigators: Iribarren, Rubinstein). In our work with patients undergoing active treatment, we identified priority components of the mobile app, such as the need for reliable TB education, medication reminders, interactivity with a treatment supporter, treatment and side effect tracking, and social networking [14]. Further modifications were completed to integrate patient recommendations of, for example, simplifying reporting steps, improving treatment progress visualization, and developing a test strip that was easier to use. The refined TB-TSTs app has the following main functionalities: (1) TB disease and treatment education, (2) treatment adherence tracking (both by self-reporting and direct metabolite test strip images), (3) tracking self-reported potential treatment side effects, (4) interactive messaging with a treatment supporter, and (5) anonymous discussion forum to connect patients with others in treatment. This trial will be conducted in Argentina where TB treatment success rates continue to be low, the default (incomplete treatment) rates are high, patients commonly receive self-administered treatment, and strong research collaborations have been established [15]. The findings will be presented and discussed with key stakeholders and policy makers to support leaders who may expand and adapt mHealth tools to meet the needs of their communities. Guided by strong theoretical frameworks [16,17] and integrating direct adherence monitoring and personalized feedback and support, this work has the potential to improve TB treatment outcomes and have a sustainable public health impact. The findings have broader implications and will improve our understanding of how to support patients in challenging treatment regimens for both communicable and noncommunicable diseases.

### Objective

The aims of this study are to (1) evaluate the impact of the TB-TSTs on treatment outcomes (success and default rates) compared with usual care and (2) assess patient and provider perceptions of the facilitators and barriers to implementation of the TB-TSTs and synthesize lessons learned.

We *hypothesize* that managing drug-sensitive pulmonary TB in adults using TB-TSTs intervention will result in improved treatment outcomes and patient satisfaction with care.

## Methods

### Trial Design

The TB-TSTs trial is a 2-arm, unblinded, pragmatic, randomized trial in which 400 participants with a confirmed diagnosis of drug-susceptible pulmonary TB initiating treatment will be randomized 1:1 into 2 parallel groups. We will follow up with patients through their full treatment course (6 months) and compare the TB-TSTs intervention with usual care. The unit of randomization will be individual patients seen at hospitals where they receive TB care by self-administered treatment. The objective of this study is to evaluate the effectiveness of the TB-TSTs intervention for improving treatment outcomes for patients (as measured by increased treatment success and reduced defaults) and as a tool to support health care professionals to more easily manage TB cases. A protocol outlining the methods of this trial was registered at ClinicalTrials.gov (NCT04221789) [18]. The trial and interventions are described in accordance with the CONSORT-EHEALTH (Consolidated Standards of Reporting Trials of Electronic and Mobile Health Applications and Online Telehealth) guidelines for reporting mobile randomized controlled trials [19].

### Ethics Approval and Consent to Participate

This study was approved by the Institutional Review Boards of the Ministry of Health of the province of Buenos Aires (ACTA-2019-15552860-GDEBA-CECMSALGP) and the University of Washington institution review board Committee in Seattle, United States (STUDY00007533).

### Theoretical Framework for Intervention Development

The information, motivation, and behavior change model was used for behavior change content and educational material development and to guide coding of qualitative focus group data during intervention development [20-22]. The intervention is guided by the theory domains through multiple mediators, including education, cues to action and skill building, and personal and social motivation elements (eg, behavioral incentives). For example, education related to strategies to remember to take medication daily corresponds to *self-efficacy behavior skills* to incorporate behavior into daily life. Education related to disease transmission and medication side effects corresponds to *adherence information*.

### Eligibility Criteria

Individuals are eligible if they are 18 years or older, newly diagnosed with drug-susceptible TB, have regular access to a smartphone, and are able to operate the phone or have someone to assist. It is estimated that by the end of 2021, 73% of Argentina’s population will have smartphones and smartphone access will continue to increase over the next 5 years [23]. Participants will be excluded if they are severely ill (ie, requiring hospitalization), reside in the same household with another study participant, or are confirmed to have drug resistance. Individuals without access to a smartphone or those who reside in areas without cellular network coverage will be excluded. Screened patients who do not meet study eligibility will have specific screening data (including sex, age, and reason for exclusion) entered into the study database to examine reasons for exclusion and feasibility of enrollment criteria.

*Case definition—*is defined as TB confirmed by positive results on a sputum smear test or diagnosis of pulmonary TB based on radiological findings and clinical signs and symptoms but with negative results on sputum smear test. The diagnosis may be confirmed by other methods, such as nucleic acid amplification (polymerase chain reaction), or enzyme-linked immunosorbent assay.

### Study Settings

The study will be conducted in the capital and the province of Buenos Aires, Argentina, where more than 65% of the annual TB cases in the country are diagnosed and treated. Most patients are at respiratory specialized public hospitals where they receive treatment by self-administration. We selected 4 public hospitals with varying geographical catchments in high TB burden areas defined by established criteria from Argentina’s National Tuberculosis Program (NTP). The study sites together treat approximately 1400 patients with TB annually. Reports from the NTP for the last 5 years and our cohort study showed that more than 60% of patients with TB were managed in public hospitals and 65% of them received treatment by self-administration. Of these, 85% were of low socioeconomic status, 50% were unemployed, 25% were smokers, and 20% reported alcohol or drug use [24].

### Participant Recruitment

**Figure 1:**
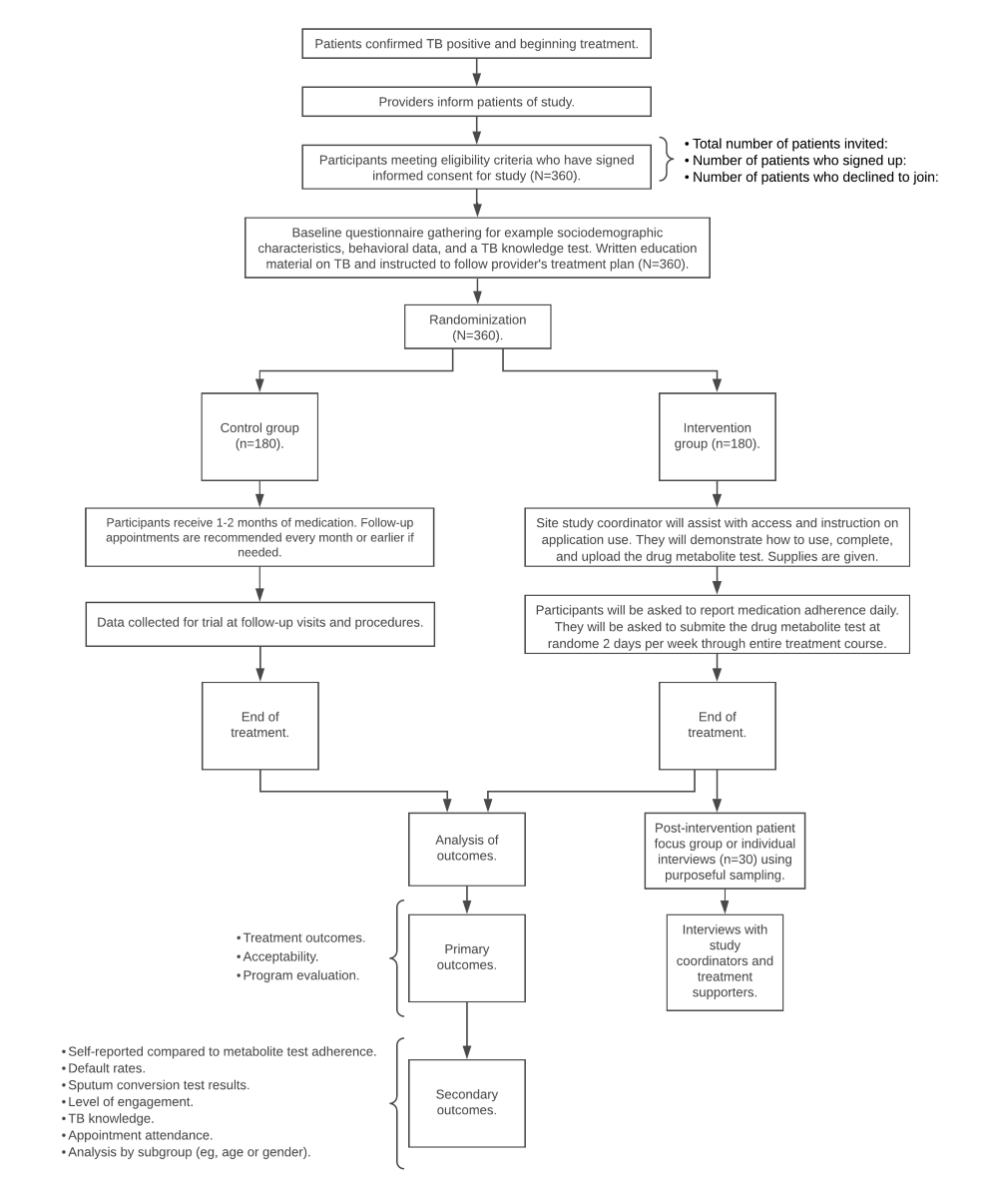
Recruitment flow diagram. TB: tuberculosis.

Patients will be enrolled on an ongoing basis, and the research team will invite every eligible patient to participate until the required sample size is achieved. The research team at each site will present the study to newly diagnosed patients as they are routed to register and receive treatment. Eligibility will be assessed by a trained clinical staff member who will then describe the study to the eligible patient face to face and answer any study-related questions. Patients interested in participating in the study will be required to provide informed consent, after which they will complete a baseline study survey that will include sociodemographic characteristics, behavioral data, self-management measures, and a TB knowledge test. A recruitment log will be maintained to document screened patients and reasons for declining participation (if willing to share reason). The primary recruitment strategy is to screen all patients who are diagnosed at the study sites within the first week of their treatment. We estimate that a total of 20-25 individuals per month will be enrolled by the research team. The COVID-19 pandemic has affected care seeking and the diagnosis of TB [25,26]. We anticipate that participant enrollment will take approximately 2 years. All participants will receive the standard TB education, including TB treatment, potential side effects, and need for treatment adherence. They will then be randomized into one of the two groups. The recruitment flow is shown in Figure 1.

### Randomization Procedures

We will randomize participants sequentially with a ratio of 1:1 allocation to receive either usual care or usual care plus TB-TSTs. A random allocation sequence will be generated using a randomization software. We will use randomly permuted blocks of different sizes between 6 and 10 and stratified by hospital to ensure that the numbers are balanced by center and group. Each block will contain equal proportions of the control and intervention groups. The randomization sequence will be uploaded to REDCap (Research Electronic Data Capture) for treatment allocation concealment by the department of data management at the Institute for Clinical Effectiveness and Healthcare Policy. Once a patient signs the consent form, they will meet with the treatment supporter who will access the REDCap randomization module to access the assigned randomization.

### Blinding

Owing to the nature of the intervention, blinding to the group allocation is not possible for participants after assignment to the study arm. The research staff allocating participants to study arms during randomization will be blinded to the sequence to minimize the likelihood that research staff will be able to predict the next study arm assignment. Clinicians will not be made aware of the group allocation unless their patient informs them. Site coordinators, who will collect the monthly standard NTP follow-up information (eg, return date to pick up medication and data from the follow-up visits) and final treatment outcomes will be blinded to the group allocation of the participants. Study investigators will be blinded to the allocation and preliminary analysis before the end of the study. Only the trial statistician will be unblinded to the analysis.

### Interventions

#### Overview

Regardless of the study arm, participants will receive standard instructions, NTP educational material, and brochures. TB treatment is provided free of charge in the public health system. The standard of care includes routine clinical and laboratory tests. Treatment of drug-susceptible TB consists of a 6-month regimen consisting of a 2-month *intensive* phase of 4 drugs (rifampicin, isoniazid, pyrazinamide, and ethambutol or streptomycin) followed by a 4-month *continuation* phase with isoniazid and rifampin daily [27]. Where available, medications are provided in a combined pill with 3 or all 4 drugs.

#### Control

At the study sites, self-administration of treatment is usual care. In general, patients receive a 1- to 2-month supply of medication and are asked to return monthly for follow-up appointments, or earlier if they experience problems. Patients in the control group will be followed up for routine visits according to the NTP guidelines. The research staff will collect data on the participants’ follow-up visits and procedures.

#### Intervention

**Figure 2:**
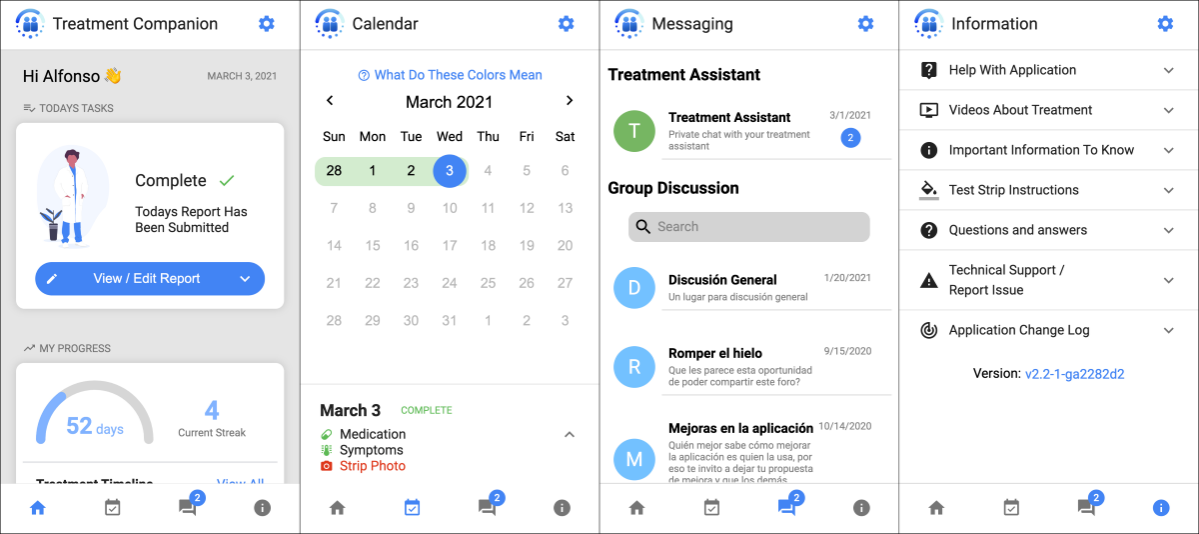
Patient app features.

TB-TSTs patient app version 2.0+ is a progressive web app that includes the following functions: (1) *TB disease and treatment education*, which is provided in written and video form and will be sent weekly as brief messages that correspond to the treatment phase; (2) *treatment progress tracking*, which includes both daily self-reporting and direct metabolite test adherence with the corresponding calendar and treatment progress indicator; (3) *potential treatment side effects reporting,* after which the treatment supporter will reach out for further assessment; (4) *interactive messaging with a treatment supporter* to ask questions and help resolve issues; (5) *medication and appointment reminder setting*; and (6) *anonymous discussion*s to connect patients with others in treatment (a request by prior patients; Figure 2).

Participants will be asked at random 1 to 2 days per week to complete a direct drug metabolite home-based urine test and upload an image of the test in the app. The test is a paper-based colorimetric test that is based on classic chemistry (the Arkansas method) that changes the color of the strip to purple when a drug metabolite of isoniazid (isonicotinic acid hydrazide; INH) is present in urine [28-30]. INH, one of the main first-line TB drugs, is considered an ideal target and good biomarker for daily adherence monitoring because its metabolites are detectable in urine for approximately 24 hours [31]. INH is often combined with other TB drugs in the same pill. Therefore, testing for INH metabolites in urine is an indicator that the combined pill was ingested within the last 24 hours. The test is quickly dipped (1-2 s) in a collected sample of urine and allowed to run for 15-20 minutes. The daily reports and test image will sync automatically to the treatment supporter interface.

At each study site, a member of the TB team (eg, a TB specialized nurse or social worker) will serve as the treatment supporter and log in to the secured TB-TSTs system via a tablet or laptop to review daily reports and test results. The treatment supporter will thus be able to track missed doses and/or reported side effects and follow up within the app with appropriate support or advice using actions specified in the study standard operating procedures. The treatment supporter will log contacting participants, determine the results of the test strip, and monitor the discussion board for group questions or concerns.

#### Treatment Supporter

**Figure 3:**
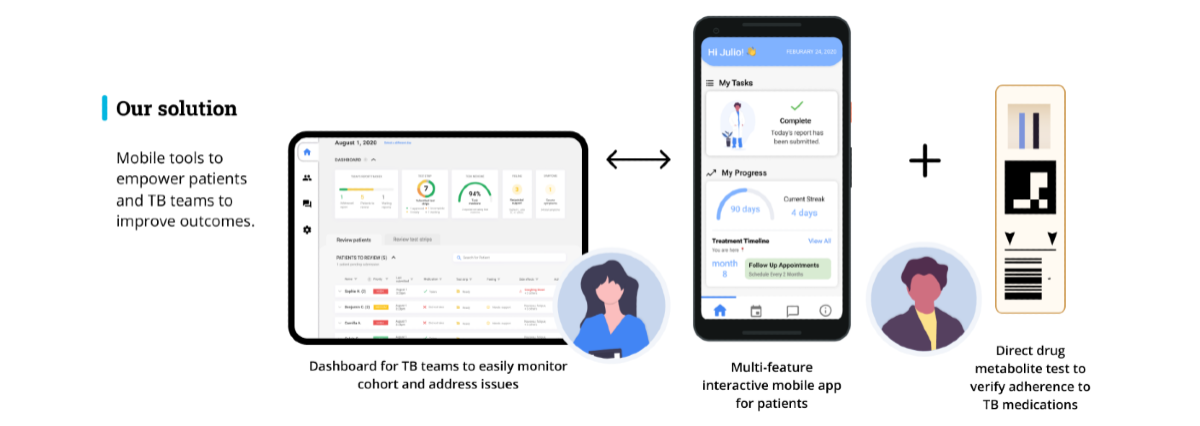
Intervention overview. TB: tuberculosis.

The treatment supporter will be trained on app and provider portal use, research goals, and protocols by the primary investigators and collaborating regional TB program team members. The main function of the treatment supporter is to oversee daily reports and help address participant questions and challenges. The treatment supporter facilitates a communication route to mitigate unforeseen events. Overall, the role is supportive to participants to create a sense of companionship and trusted guidance to improve commitment to treatment responsibilities. Figure 3 provides an overview of the TB-TSTs intervention.

### Procedures

#### Overview

TB-TSTs group participants will be assisted with gaining access to the app and will receive verbal and written instructions as well as a one-on-one demonstration of app features by the site research team member. They will also receive verbal and written instructions on how to complete the paper-based drug metabolite test and upload an image into the app (instructions also within the app). Participants in the intervention group will be informed that interaction with the treatment supporter through the app is provided within a clinic-based system during office hours and that any emergencies must be directed through standard routes.

#### Access to the App

Access is granted by the treatment supporter who enters the patient’s details to generate a one-time user password that they can use to set up the participant’s account. This code will be given directly to a patient or sent via WhatsApp (Facebook, Inc) along with a link to the web application. The treatment supporter will guide the participant through each step of the app’s installation, explain the use of the app, and demonstrate how to perform a complete report. At the first visit to the web application, they will be prompted to install it on their home screen for easier access. After installation, the app functions like any other app on a mobile phone. When a patient first logs in to the app, they will be prompted to change their password and complete an onboarding survey where we gather some of their preferences. Passwords can be updated, and new temporary passwords are provided if the password is forgotten.

#### Compensation for Expenses

Patients in the intervention arm will be asked to complete an exit survey and interview (described in the following section). We will pay for 1 GB of data per month to cover data use from the app.

### Study Outcomes

The primary outcome will be treatment success based on WHO definitions [27]. Treatment outcomes are defined as success (cure or completion of regimen), default, transferred out, deceased, or lost to follow-up. Secondary outcomes will include self-reported adherence compared with direct adherence test results (self and direct adherence), technology use measured by actual use of the app, and technology usability measured by the Health Information Technology Usability Evaluation Scale [32].

### Power and Sample Size

The trial is powered on the primary outcome measure of treatment success. Argentina’s WHO TB country report indicates that treatment success rates have ranged from 44% to 66% over the past 10 years [33]. These rates include cases that were lost to follow-up. For those with known outcomes, Argentina’s NTP and our previous cohort study estimated that the treatment success rate among patients under self-administered treatment was approximately 70%, with a default rate of 20% [34]. To detect a success rate of at least 85% in the experimental group with 90% power, the calculated sample size is 360 individuals (180 subjects per arm). Assuming an attrition rate of 10%, we would require a minimum final sample size of approximately 400 (200 per group). All calculations are based on a 2-sided test with α=.05 level.

### Statistical Methods

All analyses will be based on intention to treat in their respective intervention categories. Two-tailed tests (both chi-squared and *t* tests) will be used for dichotomous or categorical and continuous variables, respectively, to assess group assignment differences. One-tailed tests will be used to evaluate the primary outcomes. Standard descriptive statistics of frequency, central tendency, and dispersion will be used to describe each sample. A *P* value less than .05 will be set to detect a statistically significant difference for all analyses. We will compare group baseline characteristics, including age, sex, education, income, comorbidity, travel time to center, medication regimen adherence, and baseline TB knowledge. The primary end points will be evaluated using the Chi-square test. Although we do not expect group differences, logistic regression analysis will be used to adjust for potential confounders if necessary. Statistical analyses will be performed using STATA version 16. Table 1 provides an overview of the variables, hypotheses, operationalization, data collection, and measurement points.

**Table 1:** Overview of variables, hypotheses, operationalization, and data collection.

Outcome or variable				Hypothesis		Outcome measure		Analysis			Measurement point										
											Screening (T0)			Baseline (T1)			2 months (T2)			6 months (T3)	
**Primary outcomes**																					
	**Treatment outcomes**		Intervention> control; intervention<control		Treatment success (%); other outcomes (eg, failed, default, or died)		Chi-square test												✓		
		Acceptability^a^	N/A^b^		MARS^c^ scale (Likert scale), interviews		Descriptive or qualitative												✓		
		Program evaluation^a^	N/A		Feasibility, ease of use, recommendation		Descriptive or qualitative												✓		
**Secondary outcomes**																					
	Drug metabolite test^a,d^		N/A		Self-reported compared with test results		N/A									✓			✓		
	Default rate		Intervention<control		Abandon treatment for 2 months or more		Chi-square test												✓		
	Sputum conversion		N/A		At 2, 4, and 6 months		GEE^e^						✓			✓			✓		
	Global Health PROMIS^f^ SFv1.1		Intervention>control		CDE^g^ outcome for self-management		One-tailed *t* test			✓									✓		
	Level of engagement^a,d^		N/A		Daily use, message, question counts		Descriptive			✓			✓			✓			✓		
	Appointment attendance		Intervention>control		Proportion		Chi-square test						✓			✓			✓		
	**Subgroup analyses**								Chi-square test												
		Sex	Female>male		N/A		N/A														
		Age	Older>younger		N/A		N/A														
		Mobile phone access type	Personal>shared		N/A		N/A														
		Time to clinic	Less and greater than 30 minutes		N/A		N/A														

^a^Data collected from the intervention group only.

^b^N/A: not applicable.

^c^MARS: Mobile App Rating Scale.

^d^Data collected throughout trial.

^e^GEE: generalized estimating equation.

^f^PROMIS: patient-reported outcomes measurement information system.

^g^CDE: common data element.

### Methods for App Data Management

#### Overview

All data generated in the app will be sent securely over the network to a virtual machine hosted on site at the University of Washington. The virtual machine is administered by a team of health care information technology experts and requires authentication as a limited subset of users for administrative access. End users interact with a web app, which securely communicates with an application programming interface hosted on the server. The application programming interface processes user input and then stores it in a suitable database (PostgreSQL database or file server).

#### Data Viewing

To view patient reports, study treatment supporters will log in to a separate portion of the app, where they have access to the reports of only the patients registered at their site. Access control is implemented at the server level to ensure that only the appropriate actors have access to patient data. For example, a patient cannot access other patients’ information and a coordinator can only see the patients they directly supervise.

#### Data Exporting

If data need to be exported from the system, they can be queried from the database and reshaped to meet the requirements of its use. Photos can be exported as a zipped folder and do not have any personally identifiable information directly attached to them.

Considerations for this app included interoperability, support of local and remote health data storage, user-friendly data visualization, and modular development for extensible future technologies.

### Methods for Postintervention Interviews or Focus Groups

#### Overview

Following the intervention, we will conduct 3 focus groups of 8-10 people randomized to the TB-TSTs group and 2 focus groups with TB treatment supporters and TB team members to assess their experience using the TB-TST and its accompanying case management platform. Individual interviews will be conducted with those unable to attend focus group sessions. We will assess patients’ and providers’ perceptions pertaining to the facilitators and barriers to implementation of the TB-TSTs intervention and synthesize lessons learned.

#### Sample

We will attempt to include participants who abandoned treatment, those who had difficulties, and those who completed treatment successfully. Conducting 2-3 focus groups has been found to capture about 80% of themes on a topic using a semistructured guide [35].

#### Procedures

The focus groups will be 60-90 minutes in length. Following completion of the informed consent process, all focus group sessions will be audio recorded. The focus group guides will be informed by the Mobile App Rating Scale to assess *acceptability* (perceived usefulness and ease of use) [36]. The Mobile App Rating Scale has been shown to be a highly reliable tool for assessing app quality [36-38]. The scale includes 3 sections and a modifiable app-specific section. Questions include identifying challenges and bottlenecks, if the intervention meets needs, satisfaction with care, confidentiality concerns, and postintervention perceptions and recommendations. We will use a sociotechnical perspective that considers the structure process outcome to understand, for example, issues of workflow and altered practice and delivery of service and evaluation [39]. From a human-centered design standpoint, we will assess, for example, accessibility concerns, awkward flows for actions, confusing features, and user interface issues to identify the need for optimization and improvement.

Participants will be compensated the equivalent of US $20 in Argentinean pesos for their time participating in an interview or focus group.

#### Analysis

The focus group transcripts will be transcribed verbatim for coding [40]. The transcripts will be entered into a qualitative data management software, such as NVivo, to organize and facilitate analysis. We will iteratively code using thematic and descriptive qualitative methods [41,42]. Specifically, we will use an inductive approach that provides a systematic set of procedures for analyzing and deriving reliable and valid findings from qualitative data [43]. The following steps will be used for analysis: (1) preparation of raw data files—clean data; (2) close reading of text to gain an understanding of the issues discussed by the participants; (3) creation of categories (codes): identify and define categories, themes, and subthemes; (4) reconcile overlapping coding and revise coding scheme; (5) continuing revision and refinement of coding scheme: within each category, search for subtopics, including contradictory points of view and insights and selected appropriate quotes that convey the themes; and (6) apply the final coding scheme to the full data set and assess intercoder reliability.

The transcripts will be independently coded by 2 team members. The coding team will meet regularly to discuss the categories of themes and synthesize interpretations across the team. During the application of the final coding scheme, we will assess if we are applying the coding in the same way and resolve discrepancies between coders by discussing until a consensus is achieved. We will code the transcripts in the native language and translate exemplar Spanish quotes to English after coding for the final presentation. The findings will be organized into main categories of actionable change for further iterative intervention modification, considerations for broader application (or not), barriers and facilitators, and lessons learned.

#### Data Management

Data collection forms will be developed using REDCap, a secure, web-based app with interactive data capture checks designed to support data capture for research studies, providing an intuitive interface, audit trails, and automated export.

## Results

### Overview

We will present the primary and secondary findings of this study. The main findings will include treatment outcomes based on the WHO definitions and results of the intervention use, such as rates of self-reported adherence versus confirmation of adherence based on the test strip results, app use, and participant usability assessments. We will present patients’ and providers’ perceptions pertaining to the facilitators and barriers to implementation of the TB-TST intervention and synthesis of the lessons learned.

The study was approved by 2 institutional review boards and the ethics committees at each of the participating recruitment sites. Enrollment into the randomized controlled trial began in November 2020. Enrollment is expected to be completed by the end of 2022. Follow-up will continue for 6 months after the final participant is enrolled. Postintervention surveys will be carried out as soon as possible following the participant’s time in the study. Focus group sessions will begin after one-third of the participants have completed treatment. Data collection is expected to be completed 9 months after the last participant is enrolled. Results from the analyses based on the primary end points are expected to be submitted for publication within a year of data collection completion.

The findings will be presented and discussed with key stakeholders (patients, TB teams, and regional and national TB program officers). The study team will present findings to the health care professionals at recruitment sites that are open to the community to disseminate findings and encourage feedback and involvement for widespread dissemination and use and further tailoring to meet the local needs. We aim to build the capacity of leaders who may expand and adapt mHealth tools to meet the needs of their communities.

### Trial Status

Initiation of the TB-TST trial was delayed because of the COVID-19 pandemic and country lockdowns. Recruitment began in November 2020 at the first of the 4 sites.

## Discussion

We will discuss the main primary and secondary findings of this study.

### Rationale

TB remains an urgent global health threat and one of the world’s deadliest infectious diseases (above HIV). Nonadherence to treatment is a known cause of poor individual and societal outcomes, including prolonged infectivity, relapse, increased morbidity and mortality, and the development of drug resistance [44-46]. The spread of TB is exacerbated by a myriad of challenges to patients such as the lengthy treatment course, social stigma, fear, discrimination, poverty, lack of knowledge about the disease and its treatment, medication side effects, and lack of support [45,47,48]. Furthermore, growing rates of drug resistance threaten to reverse the progress made by TB eradication efforts to date [15]. It is estimated that by 2050, drug-resistant TB alone could kill as many as 2.5 million people per year and cost the global economy up to US $16.7 trillion [49]. Drug-resistant TB is more contagious, costly, and deadly. In the United States, the estimated cost to treat one case of multidrug-resistant TB is US $243,000 compared with US $46,000 for drug-susceptible TB [50]. Health care systems are burdened by the volume of patients, the HIV epidemic, lack of resources, and lack of advanced monitoring and tracking technology [51,52]. As a result, prevention of the spread of the disease and development of resistance is a global public health priority [3]. To achieve this, health care systems must ensure the completion of treatment.

The incomplete application of effective control measures has resulted in incidence and death rates associated with TB being either stagnant or decreasing more slowly than expected [53]. The current recommended target rate for treatment success is 90% for all identified cases [3]. The WHO Americas region has the lowest treatment success rate (75%), one of the highest rates of patients who are lost to follow-up, and a high percentage of deaths because of TB compared with other WHO regions [54]. In Argentina, treatment success rates have been one of the lowest in the region, from 44% to 66% since being tracked, and there have been consistently high rates of treatment default (abandoning treatment for a minimum of 2 months) of about 30% [33,34]. In addition, it is one of the 5 countries in the Americas with a high number of estimated multidrug-resistant TB cases [15]. Therefore, Argentina is one of the countries in which the health care system needs to implement more effective TB treatment adherence strategies.

Currently, there is a lack of feasible, acceptable, and effective strategies to directly monitor treatment adherence and provide timely support for patients undergoing self-administered treatment. The current strategies are recognized as insufficient to meet the goal of TB elimination in this century [3,4]. The mHealth approaches for TB treatment adherence monitoring being tested include *indirect* patient-facilitated or device-facilitated monitoring (eg, self-reporting, medication bottles containing sim card, and video observed therapy) [55,56] and *direct* monitoring through embedded sensors [57] or drug metabolite testing [28,58]. Concerns regarding the privacy, accuracy, and costs of these interventions have been raised [9]. For example, although direct video monitoring may avoid issues of stigmatization, patients can feign ingestion. Drug metabolite testing is potentially superior to video observed therapy because it requires actual ingestion of the drug and has been identified as the most promising, ethical, accurate, and least intrusive and stigmatizing of these mobile strategies, yet its potential remains largely unexplored [9]. Preliminary reports outside of the peer-reviewed literature highlight the potential for drug metabolite testing [59]; however, there is a need for further development of the technology and rigorous research to assess its impact on treatment outcomes.

In response to the challenges of implementing directly observed therapy, current developments in treatment management involve the utilization of digital health (eg, eHealth, mHealth, and connected health) technology, such as the use of cellular phones, tablets, smartphones, and other wireless devices, to explore more efficient and effective ways to ensure that patients complete their treatment [6]. For example, apps can facilitate real-time adherence monitoring or side effect tracking, on-demand or tailored education, and bidirectional communication with health care teams [10]. In a systematic review of TB-related apps in the marketplace, most of the apps targeted health care workers (eg, dosing calculations and treatment recommendations) or provided general TB information [12]. Few apps have been developed for use by patients, and none have been developed to support TB patient engagement in their own care (eg, reminders, side effects monitoring, or interaction with their health care providers), thus limiting the potential of these apps to facilitate patient-centered care. Given that more than 95% of the global population is living in an area that is covered by a mobile cellular network and mobile phone and smartphone ownership is rapidly rising, maximizing mobile tools for treatment support holds promise to heighten patients’ engagement in care and improve adherence, monitoring, communication, and delivery of evidence-based interventions [6,9,60]. However, more evidence is needed to develop, adapt, and validate these tools under diverse conditions and models of care [6,7].

This protocol describes the design and methods of a randomized controlled trial assessing the effectiveness of using TB-TSTs to improve TB treatment success rates in high TB burden setting in Argentina. This study leverages the accessible features of a mobile phone app, a direct drug metabolite test, and interactive remote support by a treatment supporter. To our knowledge, this study is the first to design a TB patient-centered mobile app using an iterative user-centered design, which has been shown to improve the quality, functionality, and engagement with patient-facing health apps. We will utilize real-time data collection and provide participants with feedback on their adherence performance in graphic and text forms. The intervention will provide bidirectional messaging, which includes personalization based on participants’ responses and results (eg, side effects and adherence reporting). Unidirectional, *push* messages can provide reminders or nudges for patients to take a more active role in their management; however, the bidirectionality may maximize the intervention potential.

The proposed study is innovative for several reasons. First, the patient-facing and treatment supporter–facing app facilitates interactivity and timely personalized support throughout the course of treatment to address problems or potential side effects. Second, the reengineering of a paper-based test for home use for detecting a drug metabolite in the urine and mobile phone image capture of the results together allow for novel real-time direct adherence monitoring. The test confirms medication ingestion within approximately the previous 24 hours, thus avoiding the poor accuracy of self-reporting. This may improve a treatment supporter’s ability to provide personalized or precision feedback to promote positive health behaviors and to ensure treatment adherence. Self-reporting or other surveillance methods are considered less accurate, increase stigmatization, or are intrusive to the patient [9]. Third, collecting repeated and longitudinal data on TB medication side effects by phone, throughout the treatment course, is novel in Argentina and may lead to new insights on side effects and treatment adherence over time. Finally, building the app and provider interface in a modular fashion using open-source software and open standards allows for future integration into health care systems (eg, eHealth records and surveillance systems) and future functionality expansion based on end user needs. A mobile optimized web app allows it to be used on any patient smartphone rather than relying on one operating system or phone type.

### Limitations

It is likely that patients will need assistance in navigating the app tool or other components, such as a test strip outside of the initial education upon enrollment. Additional instructions on how to use the tool have been developed, in video and written form, to support users. An onboarding session was developed to walk participants through the steps to use the app at first use, and it can be accessed later for review. The short videos demonstrate how to submit the daily report and include step-by-step instructions on how to conduct the metabolite test and upload a photo to the app. A third video provides an overview of other app features such as accessing education, setting appointment date reminders, individualized notification system for medication or appointments, and accessing messaging and discussion forums.

It is possible that participants may have inconsistent access to Wi-Fi or lose mobile phone services, which could delay reporting. An offline reporting feature was developed to support offline reporting and upload once a cellular network or Wi-Fi connection is re-established. There is also the potential for participants to lose or change their phones. To address this, we will document their WhatsApp number to reach out to participants if they fail to report for an extended period. If app access is lost, a link will be resent, allowing participants to log in with their previously set up username and password. In the case of a lost password, the treatment coordinator can provide a temporary password for the patient to create a new password. If participants forget to report, they will be allowed to report retroactively for the previous 3 days. Users will also be able to provide an explanation on reporting variances. If an image of the drug test strip is taken too soon, it may appear as a false negative and conflict with self-reported adherence. The treatment supporters may reach out to the participants to assess how the test was conducted.

### Harms or Unintended Effects

Research staff will monitor and take note of privacy breaches, technical problems, or unintended effects. This may include qualitative feedback from participants or observations from staff or researchers on intervention shortcomings or why people do not use the intervention as intended. The research staff will also monitor and document unintended positive effects, which can include qualitative feedback on the strengths of the system.

### Conclusions

The TB-TSTs trial is a pragmatic trial to test a novel, scalable approach to optimize TB treatment adherence and completion. According to the National Institute of Health, “the dynamic nature of adherence underscores the need to improve routine monitoring of individual’s adherence to recommended healthcare regimens, and to re-examine or develop new interventions to support adherence over the course of care” [61]. The significance of this study is at least threefold. First, it will be the first randomized controlled trial to use a home-based direct metabolite adherence test in combination with an interactive, multifeatured patient-centered app and treatment support platform. Second, the intervention has been tailored to the local setting and for Spanish speakers, for which there is a dearth of evidence. Third, we will assess the impact of the TB-TSTs and the potential for scaling the technology if the results suggest that it is effective. The success of TB-TSTs could inform us of the opportunities for treatment adherence and symptom management for various populations with complex care needs. Our long-term goal is to revolutionize patient monitoring, improve patient-provider communication, and promote self-management of treatment by optimizing and designing convenient and acceptable digital adherence tools that are tailored to their settings, in this case, Spanish speakers in Argentina.

## Appendix

Multimedia Appendix 1 Awarded grant peer-review report from NIH-NIAID [redacted].

## Abbreviations

CONSORT-EHEALTH: Consolidated Standards of Reporting Trials of Electronic and Mobile Health Applications and Online Telehealth
INH: isonicotinic acid hydrazide
mHealth: mobile health
NTP: National Tuberculosis Program
REDCap: Research Electronic Data Capture
TB: tuberculosis
TB-TST: tuberculosis treatment support tool
WHO: World Health Organization
