# Supplementary material for: Mobile Tuberculosis Treatment Support Tools to Increase Treatment Success in Patients with Tuberculosis in Argentina: Protocol for a Randomized Controlled Trial
Source: JMIR Res Protoc. 2021 Jun 21;10(6):e28094. doi: 10.2196/28094 (PMC8277351; doi:10.2196/28094)
Supplement: Multimedia Appendix 1 [file resprot_v10i6e28094_app1.pdf]

1R01AI147129-01 Iribarren, Sarah; Rubinstein, Fernando

RESUME AND SUMMARY OF DISCUSSION: This application proposes to refine and evaluate a tuberculosis (TB) patient-centered mobile health (mHealth) platform called TB-TST to monitor and enhance the treatment adherence of TB. The panel agrees that the application addresses a highly significant public health challenge in medication adherence to TB regimens and, if successful, the developed mHealth system would help the TB patients complete the treatment. The reviewers noted several significant strengths. The scientific premise is strong and well supported by the preliminary data. The investigators are excellent, and the research team is strong with a collaboration history. The research environment is outstanding. The application is innovative in redesign and implementation of test strips for home use with short reaction time, and a patient-centered mHealth system for both patient monitoring and communicating with care providers. The approach is well written and rigorous, with well thought alternative plan and usability intervention. The involvement of the end-user and care providers in mobile app design and the development of the test strip image database are additional strengths. The study timeline of the clinical trial is appropriate and well-described. However, the panel also identified some weaknesses, including a concern about the implementation challenge, and insufficient details about the usability and the quality assessment of the test strips. In addition, some reviewers raised the concern about the lack of discussion of the effect of literacy levels on the app usability, and sex as a biological variable is missing. Overall, majority of the panel remains enthusiastic for this promising and significant application and agrees that its impact on the development of mHealth technology for enhancing the medication adherence to tuberculosis regimens is very high.

DESCRIPTION (provided by applicant): The overall goal of this study is to refine the tuberculosis (TB) treatment support tools (TB-TST) intervention, which links an app developed using user-centered design principles and a paper-based drug metabolite urine test strip modified for home use, to improve treatment outcomes for patients with TB, and evaluate its impact by a RCT. TB is an urgent global health threat and the world's deadliest infectious disease despite it being largely curable. Poor medication adherence to TB regimens, along with challenges in monitoring patients and returning them to treatment, are important contributing factors to poor outcomes and the development of drug resistance. Individuals with TB face multiple barriers to good treatment adherence such as medication side effects, stigma, and lack of education about the disease and its treatment. With advances in, and proliferation of, mobile technology platforms, there is substantial interest in the possible use of mobile health (mHealth) interventions to address these challenges. Of the mHealth approaches under investigation for TB adherence monitoring, drug metabolite testing has been identified as the most promising, ethical, and accurate, and the least intrusive and stigmatizing strategy compared to other mobile solutions (e.g., video observation, “smart” medication bottles, ingestible sensor), yet its potential remains largely unexplored. Additionally, mobile applications (apps) may provide personalized treatment supervision, increase patients' self-management and improve patient-provider communication by offering more advanced functionalities for patient support and monitoring. However, available TB-related apps have not focused on patients as the end-users nor have they been fully evaluated. This proposal builds on preliminary work to 1) combine input from patients and experts to iteratively design the content, features, functionalities, and interface of the treatment support app and 2) optimize a paper-based test strip for testing the presence of isoniazid drug metabolites in urine to directly monitor adherence to treatment. The existing version of the TB-TST app has the following functionalities: it offers education on TB and its treatment, communication with a care-coordinator, tracks treatment adherence (both by self-reporting and direct metabolite test strip images), self-reports treatment side-effects, and retains patient's “diary” notes. The results from the pilot test of this version will be the starting point for this proposed study. Aims are to: 1) Refine the TB-TST intervention based on pilot study findings and apply principles of user-centered design; 2) Evaluate the impact of the TB-TST on treatment outcomes (success, default) compared to usual care; 3) Assess patient and provider perceptions of the facilitators and barriers to implementation of the TB-TST and synthesize lessons learned with stakeholders and policy makers. The primary outcome will be treatment success.

Secondary outcomes will include: treatment default rates, self-reported adherence, technology use and usability. Findings have broader implications not only for TB adherence but disease management more generally and will improve our understanding of how to support patients facing challenging treatment regimens.

PUBLIC HEALTH RELEVANCE: Tuberculosis remains one of the top ten causes of death globally despite it being largely curable. Patients face many challenges to adhere to treatment and mobile health (mHealth) interventions may address these challenges and support patients to complete their treatment. We will improve an interactive intervention based on the combined input from patients and TB experts and evaluate the intervention's impact on treatment outcomes in a randomized clinical trial. Findings have broader implications not only for TB adherence but disease management more generally and will improve our understanding of how to support patients in challenging treatment regimens.

CRITIQUE 1

Overall Impact: The goal is to provide more effective treatment strategies for TB (especially with respect to improving adherence). The work is in Argentina and Univ. of Washington. A major value is their redesign and implementation of test strips for home use with short reaction time (3-5 min.). This will help alleviate the problem of shortage of healthcare experts in this area. Another good outcome will be a database of test strip images suitable for developing classifiers by them and others. An app that goes along with the test strips will be developed and evaluated. But, there were too few details on the interventions to be developed.

CRITIQUE 2

Overall Impact: This application is responsive to PA-18-722 aiming to enhance patient adherence to patient treatment or prevention regimen. The current application seeks to implement and test a mHealth solution to enhance compliance to tuberculosis treatment in Argentina. Although direct observation treatment (DOT) has been shown to be very effective, it is resource consuming. In regions where cell phone technology has high penetrance, a phone app with bidirectional communication between caretakers and patients, couple with an effective way to verify compliance may be an effective solution to low compliance and failure to meet WHO objective with respect to TB control. The application is clear, easy to follow and proposes to expand on the development of an app that has already been developed, and partially field tested. Compliance is tested using a calorimetric assay on a strip testing the presence of isoniazid with automatic readout by the cell phone app. A randomized trial of 360 subjects will examine whether the intervention impact the rate of cure at six months. A third Aim will determine usability of the app. Significant preliminary work support feasibility of the project, including existence of preliminary app, filed data as to produce a better version of the app, and demonstration of the ability to enroll subjects with a new diagnosis of tuberculosis. Improved compliance to TB treatment is definitely a high priority goal. The investigators are well-qualified, operate in a supportive environment, and are fluent in Spanish. The project introduces several innovations, from the ability of the application to communicate to communicate with electronic health records to the development of a more usable drug assay. The approach has several strengths including end-user and care providers' input in app design, the provision of alternative strategies to the most common pitfalls encountered in the design of mHealth interventions, a formal usability intervention, and local authorities buy-in. There also a few weaknesses to the application including representativity of the study sample as a cell phone with camera is required, the use of a yet unproven and unstandardized test strip for compliance detection, and probable underpowering of the clinical study. Overall, the strengths and impact significantly outweigh the weaknesses thus generating significant enthusiasm for the application.

CRITIQUE 3

Overall Impact: The overall goal of this proposal is to develop tuberculosis treatment support tools named TB-TST which include an app and a paper-based drug metabolite to monitor adherence and improve treatment outcomes. They plan to refine the existing TBST with a patient centered design, perform an RCT to evaluate in impact on users vs non-users and assess patient provider satisfaction. Overall it is a well written and structured grant involving a significant health problem in both developing and under developed countries. The challenge to monitor the adherence regime is high to due to several reasons including the stigma associated with this disease. The scientific premise is based on the evidence that mHealth based system supported by education, drug usage monitoring by biochemical means along with health care provider support can improve outcomes. This proposal has several innovative aspects that result in an integrated system built on patient centered design. The investigative team is strong and is collaborative although implementation can be challenging over adherence issues which reduces the enthusiasm in a minor fashion for a strong grant proposal.
